# Molecular modeling study of micro and nanocurcumin with in vitro and in vivo antibacterial validation

**DOI:** 10.1038/s41598-023-38652-2

**Published:** 2023-07-28

**Authors:** Amal S. Othman, Israa M. Shamekh, Mohnad Abdalla, Wafa A. Eltayb, Nashwa A. Ahmed

**Affiliations:** 1 Department of Microbiology, Faculty of Applied Medical Sciences, October 6 University, P.O. Box 12585, El-Giza, Egypt; 2 Chemo and Bioinformatics Lab, Bio Search Research Institution, BSRI, Giza, Egypt; 3 Pediatric Research Institute, Children Hospital, Shandong University, Jinan, 250022 Shandong China; 4 Biotechnology Department, Faculty of Science and Technology, Shendi University, Shendi, Nher Anile Sudan

**Keywords:** Computational biology and bioinformatics, Microbiology

## Abstract

Repurposing natural compounds as inhibitory targets to combat bacterial virulence is an important potential strategy to overcome resistance to traditional antibiotics, in the present study, the antibacterial activity of micro-curcumin and nano-sized curcumin was investigated against four predominant bacterial pathogens, namely, *Escherichia coli, Pseudomonas aeruginosa, Staphylococcus aureus,* and *Bacillus subtilis.* Curcumin bactericidal susceptibility could be summarized as the order, *P. aeruginosa* > *B. subtilis* > *S. aureus* > *E. coli*. Molecular docking analysis was conducted to confirm the impact of curcumin on the most vital and positively identified quorum-sensing pathway signaling proteins SecA-SecY, LsrR, PqsR (MvfR), AgrA which act as key players in the bacterial communication systems. The in silico physicochemical properties revealed that curcumin as a nutraceutical can be classified as a drug-like compound. An in vivo infected wound model was employed in four groups of albino rats. Topical application of nano-curcumin lotion showed a marked reduction in wound area (98.8%) as well as nearly 100% reduction in total bacterial viable count compared to the control group, on the fifteenth day post-treatment post-injury. The obtained data suggested that curcumin nanoparticles exhibited superior antibacterial activity and may possess clinical utility as a novel topical antimicrobial and wound healing agent.

## Introduction

Recent spreading of microbial infections, and emergence of new pathogens including multidrug resistant species are considered humanity challenges that brings a need of alternative therapies^1^. Public interest in traditional herbs has developed due to their confirmed medical properties and limited or no side effects^2^. *Curcuma longa* (common name: Turmeric) is a rhizomatous herbaceous perennial flowering plant of the ginger family (Zingiberaceae), it is typically used for spices and as coloring agent^3^. Curcuminoids are the non-toxic, naturally occurring phytochemical polyphenol components of turmeric it contains three different structures: (a) diferuloylmethane or curcumin (curcumin I, 75%), (b) dimethoxy curcumin (curcumin II, 20%), and (c) bisdemethoxycurcumin (curcumin III, 5%)^4^. Pharmacologically curcumin has shown an extensive range of promising biological and therapeutic actions like having anti-bacterial activity against a wide range of bacteria via targeting the bacterial cell membrane, cell wall, protein, DNA, and other cellular structures, or by inhibiting bacterial growth through the quorum sensing (QS) system/pathway^5^, in addition its anti-inflammatory^6^, anti-cancer^7^ as well as anti-diabetic actions^8^ were proved.

Pharmacokinetically curcumin is not soluble in water, unstable in solutions, and shows low bioavailability, poor absorption, and rapid elimination from the body^9^. These drawbacks hinder its in vivo use as a therapeutic agent. To overcome these problems, it is necessary to design an advanced drug delivery system that can enhance the therapeutic translation of curcumin^10^. Several studies have concluded that the nanosizing of curcumin particles can have better hydrophilicity, chemical stability, sustained release and adequate dispersibility of curcumin compared to its free form^11^.

Several studies have indicated that curcumin therapy, especially if administered topically promotes wound healing by shortening healing time, enhancing collagen deposition, and increasing skin fibroblast and vascular density, being a wound healing proangiogenic agent in both healthy and infected wounds^12^. Since curcumin is a well-known drug, molecular docking in that context can be performed either for investigating the binding capacities of curcumin with novel targeted molecules or for curcumin analogs. Quorum sensing (QS) microbial communication system is used by a variety of bacterial stains^13^, it relies on the generation and detection of signaling molecules (self-inducers) to monitor population density^14^. Most Gram-negative bacteria use *N*-acylhomoserine lactones as the major QS signal molecules, while Gram-positive bacteria use signal peptides to regulate physiological functions, such as biofilm formation, biosynthesis of antibiotics, bioluminescence, release of virulence factors, siderophore, extracellular proteases, and cell motility^15^. Curcumin plays an important inhibitory role through the bacterial QS system as it interacts with numerous molecular targets and transduction pathways through a multi mechanism anti-infection strategy^16^. There are multiple proteins involved in the quorum sensing pathway, SecA–SecY, LsrR, PqsR (MvfR) and AgrA proteins related to* Bacillus subtilis*, *Escherichia coli*, *Pseudomonas aeruginosa* and *Staphylococcus aureus* respectively were found to be the most vital and positively identified QS regulators, they are integral parts in the QS network and act as key players in the bacterial communication systems, SecA–SecY channel protein, permits a wide range of bacterial proteins to be transported across the eukaryotic endoplasmic reticulum membrane or across the prokaryotic plasma membrane^17^, LsrR protein regulates hundreds of genes that participate in myriad biological processes, including mobility, biofilm formation, and antibiotic susceptibility as well as bacterial resistance to various compounds^18^, PqsR (MvfR) is a critical transcriptional regulator that can increase antibiotic efficacy and eventually prevent the resistance proteins from forming^19^, moreover, AgrA transcription factor protein is involved in the regulation of the quorum-sensing response via generation of hemolysins and other virulence factors^20^. Finally curcumin was identified as a potent QS inhibitor of the human pathogens that would not induce the bacterial resistance as this blocking strategy has been considered as substitutional to traditional antibiotics^15^.

The present study aimed to explore the antibacterial effect of micro and nano curcumin on significant bacterial pathogens, molecular docking and screening of ADME/drug-likeness properties by using in silico pharmaco-informatics approach for curcumin were presented, moreover the feasibility of the In vivo wound healing activity of the lotions prepared from micro and nano curcumin was checked to validate and prioritize their biological effects.

## Materials and methods

### Chemicals

Micro-curcumin (MC), dichloromethane, Nutrient agar, Muller Hinton agar, glycerol, phosphate-buffered saline, white paraffin, petroleum jelly, ketamine, Soframycin ointment and Ciprofloxacin, purchased from Sigma Chemicals Co. (USA). Experimental research on used plants complied with relevant international guidelines and legislation.

### Preparation of curcumin nano-sized particles

Nano-sized curcumin (NC) was prepared by solvent–antisolvent precipitation, in which 100 mg of micro-curcumin was suspended in 20 mL dichloromethane (solvent), 1 mL of this solution was added to 50 mL boiling water dropwise with a flow rate of 0.2 mL/min under ultrasonic conditions (ultrasonic power of 100 W and a frequency of 30 kHz). After sonication for 10 min, the contents were stirred at room temperature for 20 min (200 rpm) until we obtained a clear, orange-colored solution. The solution was concentrated under reduced pressure at 50 °C and was then freeze-dried to obtain a pale orange powder which was scanned under the electron microscope to ensure its particle size. The water solubility of both MC and NC was compared^21^.

### Characterization of nano-curcumin (NC)

UV–visible spectroscopy (Genway-Australia spectrometer) was used to characterize NC particles, the absorbance was scanned from 200 to 700 nm, the mean particle size was investigated using Dynamic light scattering (DLS) in which 1 mg of dried powder was suspended in 10 mL sterile distilled water, the morphology of the particles were examined using transmission electron microscopy (TEM, Jeol JEM-1400, Japan) in which a drop of the plant solution was placed on the carbon-coated copper grids and dried by allowing water to evaporate at room temperature. Electron micrographs were visualized at 70 kV. All experiments were done at The Regional Centre for Mycology and Biotechnology (RCMB) Al- Azhar University, Egypt^22^.

### Bacterial strains and maintenance procedure

The international reference Gram-negative bacterial strains *Escherichia coli* (*E. coli*) (ATCC 25922) and *Pseudomonas aeruginosa* (*P. aeruginosa*) (ATCC 9721) and Gram-positive strains *Staphylococcus aureus* (*S. aureus*) (ATCC 25923) and *Bacillus subtilus* (*B. subtilus*) (ATCC 6633) www.ATCC.org were kindly provided by the Faculty of Pharmacy, Cairo University, Cairo, Egypt. Bacterial cultures were stored as frozen stocks within 15% glycerol at − 80 °C, prior to performing experiments. Stock strains were sub-cultured on nutrient agar plates^23^.

### Anti-bacterial activity screening

Fresh bacterial cultures of the currently tested strains were prepared, standardized suspensions were serially diluted. The test tubes were shaken thoroughly, and the turbidity of bacterial suspensions was adjusted at 10^7^ colony-forming unit/ml (cfu/mL) equivalent to 0.5 MacFarland standard tube. The antibacterial activity of MC and NC was screened against the four reference tested microorganisms using the agar well diffusion technique^24^. 100 μL (10^6^ cfu) of the bacterial suspension was added onto Muller Hinton agar plates and evenly spread, 6-mm diameter wells were punched in the agar media and filled with 100 μL (800 μg/mL in DMSO) of the tested compounds^25^. The plates were kept at room temperature for 1 h and then incubated at 37 °C for 24 h. The antimicrobial activities were evaluated by measuring the clear inhibition zone diameters. Ciprofloxacin was used as a positive reference standard to determine the sensitivity of the strains. DMSO (negative control) gives negative inhibitory results in preliminary experiment.

### Determination of total viable count (TVC)

A total of 250 µg/mL concentration of MC and NC was incubated with a standard solution of the four tested bacterial strains for 12 h. Bacterial growth was measured at 600 nm using Nano Drop-1000 v 3.3.1 spectrophotometer (Nanodrop Technologies, Inc., Wilmington, USA). Control tubes were also maintained and the TVC indicating the number of bacteria that survived after applying MC or NC, was enumerated by the plate count method. The TVC was represented as cfu/mL × 10^7^^26^.

### Determination of the minimum inhibitory concentrations (MICs) of micro and nano-curcumin

MICs of both MC and NC were determined using two-fold serial dilutions in Mueller–Hinton broth medium^27^. Individual stock solutions of MC and NC were prepared in DMSO in a concentration of 500 µg/mL, an array of serial dilution tubes were prepared each containing 500 µL of the broth media, 500 µL of the prepared plant solution was added to the first tube and the solutions were doubly diluted so that the concentrations range were from 250 to 7.8 µg. 500 µL of a standard bacterial solution (10^6^ cfu/mL) was added to each tube, Ciprofloxacin was used as positive control. The tubes were incubated for 24 h at 37 °C, the antibacterial activity was assessed at 600 nm using ND-1000v 3.3.1 spectrophotometer (Nanodrop Technologies, Inc., Wilmington, USA), the lowest concentration required to inhibit the microbial growth was regarded as MIC.

### Molecular docking studies

#### Software

The standard molecular docking analysis was carried out using SAMSON 2020 software https://www.Samsonconnect.net (trial version), discovery studio visualizer https://www.3ds.com/products-services/biovia/3ds-com/products/molecular-modeling-simulation/biovia-discovery-studio/visualizer and MOE software https://www.chemcomp.com/products.htm, as well as swissADME http://www.swissadme.ch/.

#### Ligands and receptors preparation

The 3D ligand structures Curcumin (PubChem CID 969516) https://pubchem.ncbi.nlm.nih.gov/compound/Curcumin and ciprofloxacin (pubchem CID 2764) https://pubchem.ncbi.nlm.nih.gov/compound/2764 their sub-structural characteristics were carefully selected from the literature and downloaded independently from PubChem https://pubchem.ncbi.nlm.nih.gov/ in SDF format, then transformed into MOL2 format using open Babel software http://openbabel.org/wiki.

2D/3D representations of structures of the selected compound were converted to Simplified Molecular-Input Line-Entry System (SMILES) notations and submitted to the swissADME online server http://www.swissadme.ch/ for calculation and knowledge about structure features. The swissADME was used to identify physicochemical characteristics as well as predict absorption, distribution, metabolism, and excretion (ADME) parameters, the drug-like nature, physicochemical properties of the compounds^28^. Now, the two chemicals (curcumin and ciprofloxacin) are ready to dock with the four most vital and positively identified bacterial quorum-sensing proteins obtained from the protein data bank (PDB) https://www.rcsb.org under accession numbers 6itC, 4l5J, 6yiZ, and 4g4K for the isolates *B. subtilis, E. coli, P. aeruginosa*, and *S. aureus,* respectively, all water molecules and ligands were removed, while hydrogen atoms were added to the target proteins. The docking system was created via SAMSON 2020 software https://www.samson-connect.net/ (French Institute for Research In computer science and Automation (INRIA), France).

#### Molecular docking

This technique was used to estimate the binding modes and affinities of each chemical by docking the structures of the four bacterial QS pathway proteins of accession numbers 6itc, 4l5j, 6yiz and 4g4k. The docking program operates in such a way that it can obtain the docking parameter in MOE software and SAMSON 2020 software, a visual representation of the docked poses of high-scoring compounds was often necessary because many of the ligands were docked in a variety of different directions^28^.

### Physicochemical properties

Certain physicochemical characteristics must be observed to classify the chemical as a drug^29^. including the bioavailability of absorption, the volume of distribution, and the half-life for ADMET, molecular weight (MW, g/mol), logarithm of the partition coefficient (log p), number of hydrogen bond acceptors (HBA), number of hydrogen bond donors (HBD), number of rotatable bonds (ROT), and the topological polar surface area (TPSA, Å^2^). SwissADME was used to calculate the pharmacokinetic properties of curcumin, percentage of absorption (%abs) was calculated by using the formula presented by Mitra et al.^30^.

$$\% abs = 109.9 - \left( {0.345 \times TPSA} \right)$$

### Toxicity prediction

It is important to predict the ligand examined interaction with other body proteins, to ensure that this ligand is safe, with no carcinogenic effect, therefore, in silico toxicity prediction for the curcumin ligand was constructed using the PreADMET web server for PreADME/Tox for toxicity and ADME as well as drug Likeness prediction^30^ https://preadmet.webservice.bmdrc.org/. Toxicity is measured as the Ames test, carcinogenicity on different animals, and hERG (human ether-à-go-go-related gene cardiac potassium channel) ion channel inhibition, being an important anti target in drug discovery as it is associated with potentially fatal heart conditions^31^.

### Molecular dynamics (MD) simulations

The structures of the best-docked complex for each protein are selected for in-depth molecular dynamics simulation (MDS) study for a period of 100 ns. NAMD software was utilized to conduct the MDS with CHARMM 36 force field^32^. VMD is used to prepare complexes for the MDS. Complexes are subjected to equilibration using the CHARMM GUI web server after that a production run for 100 ns. The equilibration is done on the protein-small molecule solvated in the TIP3P water model and 0.154 M NaCl solution at 310 K temperature and pH 7^33^. VMD is utilized in trajectories analysis, while the Chimera software of UCSF is used for cluster analysis^30^. After trajectory clustering, the five most populous clusters are represented by a conformation and tested for its binding to the protein. AutoDock Vina software is used in the binding energy calculations using 40 Å × 40 Å × 40 Å box dimensions^34^. The protein data base (PDB) files from https://www.rcsb.org/ of the examined bacterial proteins (6ITC, 4L5J, 6YIZ, 4G4K) were used for the quorum-sensing signaling proteins/peptides (SecA-SecY complex, LsrR, PqsR (MvfR), AgrA), respectively. The MD simulation was run on Desmond with default protocols https://www.schrodinger.com/products/desmond, and the TIP3P model in the Desmond System Builder tool was used to solve the protein. Periodic boundary conditions with a 10 Å orthorhombic box were used on the outer protein surface, and 0.15 M NaCl was used to neutralize the simulation system. The simulation was run at a temperature of 310 K and pressure of 1.013 bar for 100 ns. The trajectory was analyzed by Desmond, VMD to set up simulation systems and to view trajectories^35^.

### Assessment of the in-vivo wound healing potentiality

#### Animal ethical considerations

Animal experiments were approved by the October 6 University committee (Approval number 20180901), and performed according to relevant guidelines and regulations regarding Animal Research also Reporting of In Vivo Experiments (ARRIVE) guidelines https://arriveguidelines.org/.

#### Preparation of micro and nano-curcumin topical lotions

Each of Micro and Nano-curcumin herbal lotions was prepared by stirring each plant powder with a mixture of soft white paraffin and petroleum jelly (5% W/W). The soft white paraffin and petroleum jelly were used as a hydrocarbon base^36^.

#### Infected wound model

A total of 24 male albino rats (200–250 g) were housed in standard plastic cages with a 12 h light/dark cycle, wheat straw was used as bedding material and the temperature was maintained at 24 °C ± 2 °C. All animals had free access to food and water. The animals were anesthetized via ketamine with a dose of 50 mg/kg^37^. The dorsal thoracic central region was shaved by an electric clipper with a diameter of 3 cm. Skin wounds were created with the help of surgical blades, fresh bacterial suspension of *P. aeruginosa* on nutrient agar broth was incubated. At the log phase of growth, the suspension centrifuged for 15 min, the supernatant was discarded, and the bacteria were diluted to 10^8^ cfu/mL in 1 mL sterile Phosphate-Buffered Saline. The wounds were cleaned with sterile cotton swabs soaked in 70% ethyl alcohol. Ten µL of the bacterial suspension (10^6^ cfu) was inoculated into the rat wounds using a micropipette and smeared uniformly with the micropipette tip^38^. The duration of inoculation to successful model was 48 h, topical treatment of the two herbal lotion formulations (MC and NC) are being compared to the standard antibiotic Framycetin (Soframycin) ointment, all applied at the same size once daily and repeated for 15 days^39^.

#### Animal grouping

The animals were allocated in four groups (six animals in each) as follows:

- Group I: infected wounded animals treated with saline (negative control group).
- Group II: infected wounded animals treated with Micro-curcumin lotion.
- Group III: infected wounded animals treated with Nano-curcumin lotion.
- Group IV: infected wounded animals treated with standard antibiotic Framycetin (Soframycin) ointment (positive control group).

#### Wound healing analysis

All infected wounds were photographed from a standard height on days 0, 2, 6, 11, and 15 post-injuries post-treatment. Skin contraction, which mainly reflects wound healing, was studied by tracing the raw wound area (in cm) on transparent paper, till wounds almost completely heal, via being covered with skin epithelium. Wound healing percentage (WH %) was calculated as follows^39^:

$${\text{WH }}\% = \left( {\left[ {{\text{Initial wound area}}{-}{\text{wound area on specific day}}} \right]/{\text{Initial wound area}}} \right) \times 100$$

#### Wound Bacterial Count

A part of each wound (5 mm in diameter) was cut aseptically using punch biopsy forceps. Specimens were homogenized in 1 mL phosphate-buffered saline and centrifuged at 3500 rpm for 5 min. The supernatant was collected, and the total viable bacteria were counted on days 0, 2, 6, 11, and 15 post-treatments.

### Statistical analysis

All experiments were replicated at least three times and the statistical significance of each difference observed among the mean values was determined using the mean, standard deviation, and analysis of variance. ANOVA test was used for comparison among different times in the same group in quantitative data by (IBM SPSS Statistics for Windows, Version 20.0. Armonk, NY: IBM Corp.)

## Results

### Characterization of curcumin nano particles

**Figure 1:**
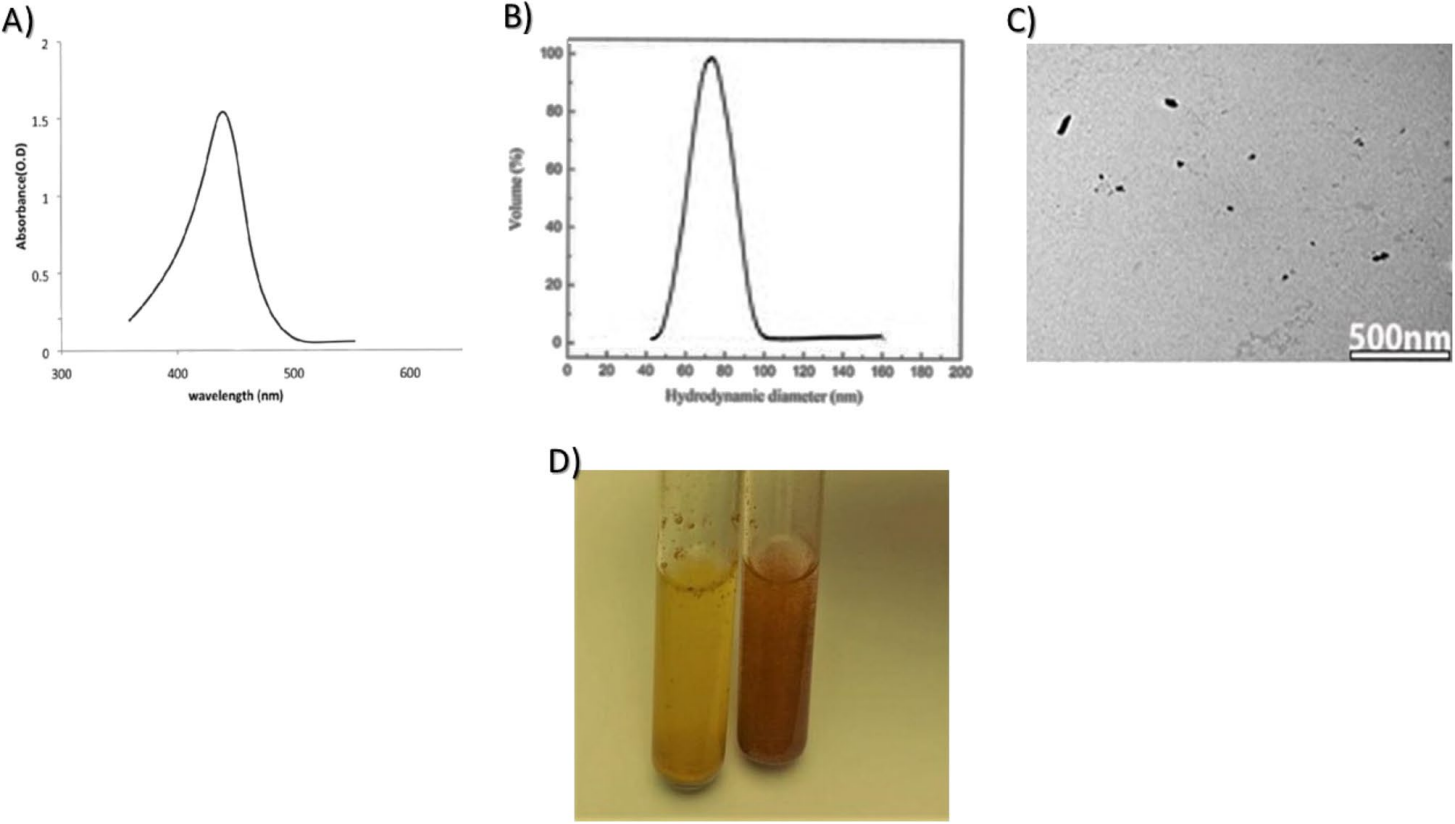
Characterization of nanocurcumin showing: UV–visible spectrum (**A**), DLS showing particle size (**B**), TEM image (**C**), and solubility of micro (yellow, left) and nano (orange, right) curcumin in water (**D**).

UV–visible spectrum indicated successful preparation of nanocurcumin showing characteristic peak at 438 nm (Fig. 1A), DLS revealed an average hydrodynamic diameter of 78.6 ± 8 nm (Fig. 1B), direct TEM visualization showed NC particles with irregular spherical shape scattered or arranged in aggregates (Fig. 1C), it was found that nanocurcumin showed better aqueous solubility than micro curcumin (Fig. 1D).

### Antibacterial assay

**Figure 2:**
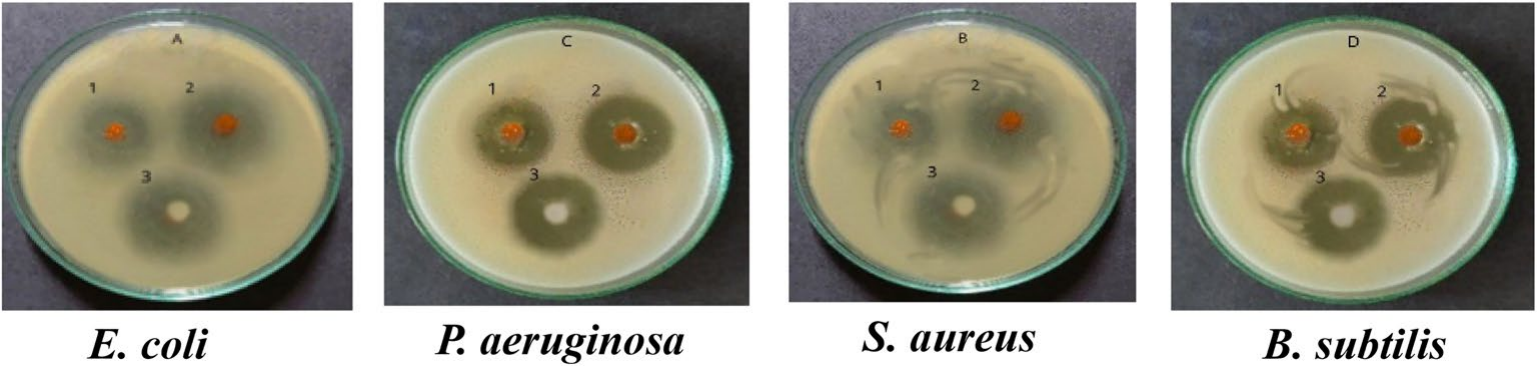
Antibacterial activity of MC (1), NC (2) and ciprofloxacin (3) against the four tested bacterial isolates.

The in vitro antibacterial effect of both MC and NC was screened against standard Gram-negative and Gram-positive strains of the most prevalent bacterial pathogens, namely *E. coli*, *P. aeruginosa*, *S. aureus*, and *B. subtilis*. Results in Table 1 and Fig. 2 showed that there were statistically significant differences between the means of inhibition zone diameters within the four bacterial groups (*P* < 0.001), the inhibition zone diameters of NC were larger than that of MC for the tested bacterial isolates indicating its higher antibacterial activity, *P. aeruginosa* showed significantly larger inhibition zone diameters for both.

**Table 1:** Inhibition Zone Diameters of MC and NC against the four tested bacterial isolates; *E. coli*, *P. aeruginosa, S. aureus and B. subtilis* in comparison to ciprofloxacin control antibiotic.

Compounds	Inhibition zone diameter (mm)				f	*p*
	*E. coli* Mean ± SD	*P. aeruginosa* Mean ± SD	*S. aureus* Mean ± SD	*B. subtilis* Mean ± SD		
MC	13.16 ± 0.1155	16.36 ± 0.12	14.43 ± 0.152	15.73 ± 0.11547	551.581	< 0.001
NC	20.56 ± 0.15275	25.5 ± 0 .10	21.29 ± 0.1049	23.10 ± 0.1001	1072.795	< 0.001
Ciprofloxacin	26.03 ± 0.11547	23.86 ± 0.75	26.2 ± 0.360	24.5 ± 0.43589	17.675	0.001

**Figure 3:**
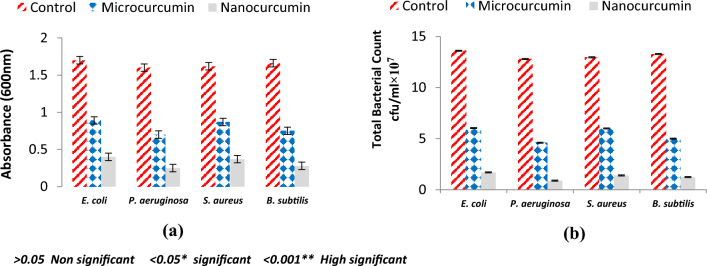
Antibacterial activity of MC and Nc based on growth turbidity (**a**) measurement and total viable bacterial count (**b**).

Broth turbidity revealing positive bacterial growth was measured spectrophotometrically (600 nm), results summarized in Fig. 3 showed that both curcumin compounds (MC and NC) showed significant bactericidal activity on the four tested bacterial strains, curcumin bactericidal susceptibility could be in the order, *P. aeruginosa* > *B. subtilis* > *S. aureus* > *E. coli*, determination of TVC emphasized this trend.

### Determination of the minimum inhibitory concentrations of MC and NC

The results presented in Table 2 summarized the detected MIC values for both MC and NC against the tested bacterial isolates. The MIC of MC ranges from 125 to 250 µg/mL, decreasing the size of the particles to the Nano range increases its efficiency against bacteria leading consequently to decrease the MIC values to be 15.6 µg/mL for *P. aeruginosa* and *B. subtilis* while it was 31.25 µg/mL for *S. aureus* and *E. coli*. The obtained MIC values were compared to the standard antibiotic which inhibits all the tested strains at 7.8 µg/mL.

**Table 2:** MIC values of MC and NC against the tested bacterial isolates.

Compounds	Minimum inhibitory concentration values (µg mL^−1^)			
	*P. aeruginosa*	*B. Subtilis*	*S. aureus*	*E. coli*
Micro-curcumin	125	125	125	250
Nano-curcumin	15.6	15.6	31.25	31.25
Ciprofloxacin	7.8	7.8	7.8	7.8

### Ligand–protein docking

**Figure 4:**
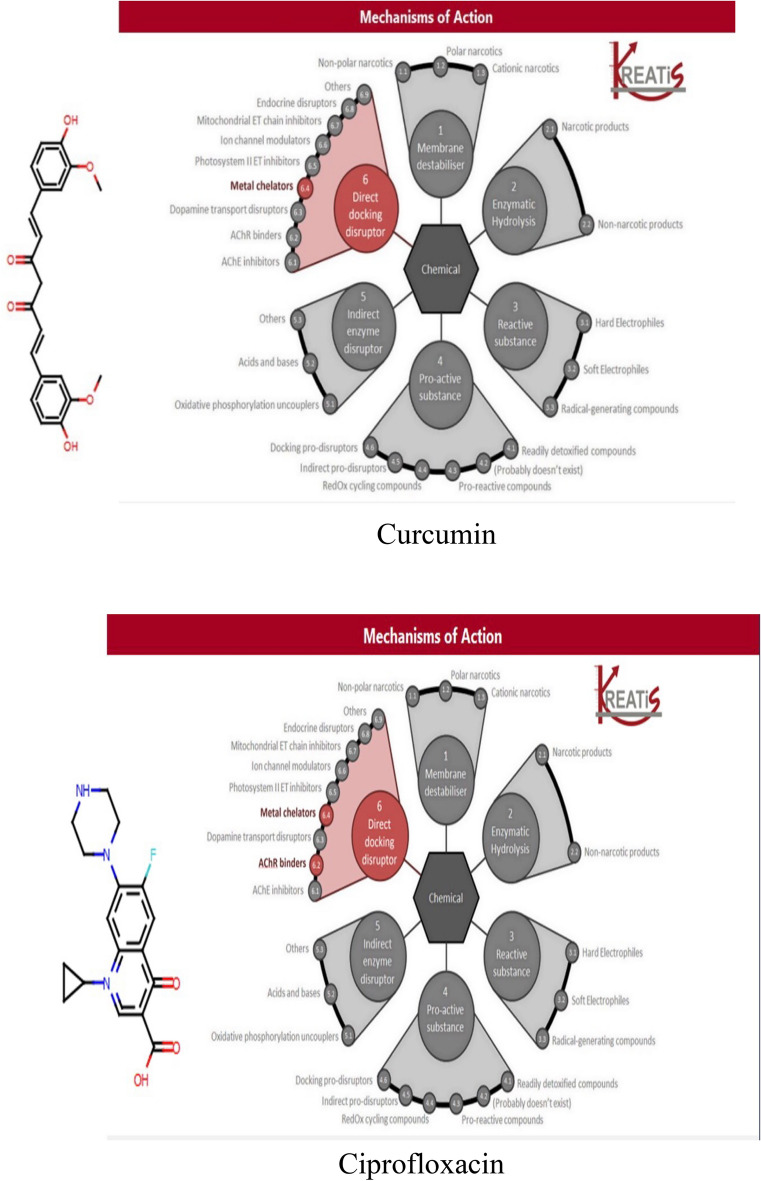
Mode of action of curcumin and ciprofloxacin.

The molecular structures of ligands curcumin and ciprofloxacin were applied in SAMSON2020 software to know the mode of action and the efficient mechanisms of action (MechoA) scheme to determine the MechoAs of the parent substance and its major metabolites, if they were identified, by their conical simile, which was retrieved from the pubcem data bases (PubChem ID for curcumin is CID 969516 and Ciprofloxacin is CID2764), the obtained results indicated that curcumin only interacted by direct docking disruptor in metal chelators, while ciprofloxacin interacted by direct docking disruptor in metal chelators and AChR binders (Fig. 4). The resulting scores for these bacterial proteins were presented in Table 3. Curcumin and ciprofloxacin ligands interacted with the bacterial protein's active site residues with energy binding affinity that varied from − 4.3 to − 7.8 kcal/mol. Where *P. aeruginosa* responded the most, to the inhibitory effect of curcumin, via inhibiting the active site of the PqsR protein with binding affinity of − 7.8 kcal/mol. Curcumin interaction results of SecA–SecY protein (*B. subtilis*) and AgrA protein (*S. aureus*) indicated moderate binding affinity − 6.6 and − 6.0 kcal/mol, respectively.

**Table 3:** Molecular docking scores of the bacterial quorum sensing pathway proteins SecA-SecY, LsrR, PqsR (MvfR), AgrA interaction via curcumin and ciprofloxacin, as the docked compounds.

Bacterial protein	Organism	PDB ID	Ligand compound	Binding affinity (kcal/mol)
SecA-SecY	*B. subtilis*	6itc	Curcumin	− 6.6
			Ciprofloxacin	− 5.5
LsrR	*E. coli*	4l5j	Curcumin	− 5.5
			Ciprofloxacin	− 4.3
PqsR (MvfR)	*P. aeruginosa*	6yiz	Curcumin	− 7.8
			Ciprofloxacin	− 6.4
AgrA	*S. aureus*	4g4k	Curcumin	− 6.0
			Ciprofloxacin	− 5.11

**Figure 5:**
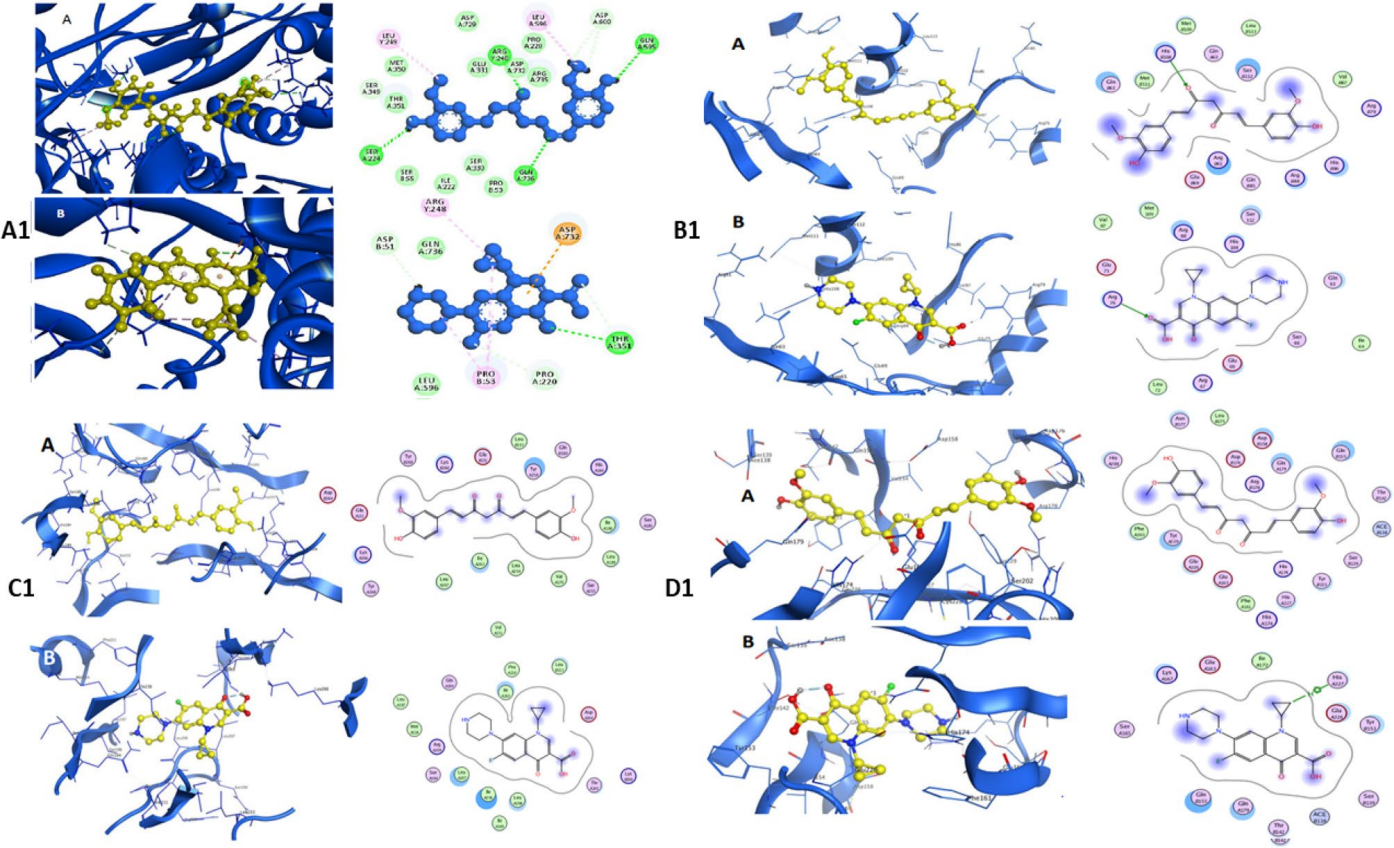
Receptor binding domain interaction with curcumin (**A**) and Ciprofloxacin (**B**), the right figure shows the 3d structure of the active site for the protein with curcumin and Ciprofloxacin, the left figure shows the 2d interacting residues of the protein with curcumin and Ciprofloxacin (**A1**): SecA-SecY protein of *B. subtilis*. (**B1**): LsrA protein of *E. coli*, (**C1**): PqsR (MvfR) protein of *P. aeruginosa*, (**D1**): AgrA protein of *S. aureus*.

On the other hand, curcumin binding affinity to LsrR protein (5.5 kcal/mol) represented week interaction. The receptor-binding domain (RBD) interaction between bacterial proteins and ligands (curcumin and ciprofloxacin) are presented in Fig. 5. The interaction between the SecA-SecY protein and curcumin is placed at the amino acid positions: Leu249, Arg248, Gln736, Asp600, Leu596, Gln595, Ser349, and Ser224 (Fig. 5A1–A), while ciprofloxacin is located at the amino acid positions: Pro 53, Pro 220, Thr 351, Leu 596, Asp 51, Gn 736, and Asp 732 (Fig. 5A1–B). The LsrA receptor-binding domain interaction between the bacterial protein and curcumin is located at amino acid positions: Leu 113, Ser 112, Met 109, His 108, and His 86 (Fig. 5B1–A), while, ciprofloxacin interaction is located at amino acid positions: Val 87, Met 109, Arg 88, His 108, Ser 112, Glu 75, Arg 79, Leu 72, Arg 67, and Glu 69 (Fig. 5B1–B). The PqsR (MvfR) protein found in P. aeruginosa has an interaction with curcumin at the amino acid positions: Lys 266, Tyr 258, Leu 254, Leu 189, Ile 186, His 184, Val1 70, and Glu 151 (Fig. 5C1–A). However, ciprofloxacin has an interaction at the amino acid positions: Gln 194, Arg 209, Met 224, and Leu 197 (Fig. 5C1–B). Finally, the AgrA protein found in S. aureus has an interaction with curcumin at the amino acid positions: Tyr 229, Cys 228, His 227, Glu 226, Phe 203 Ser 202, His 200, Gln 179, His 174, Glu 163, and Phe 161 (Fig. 5D1–A) and ciprofloxacin has an interaction at the amino acid positions: Arg 178, Asn 177, Asp 176, Leu 175, Asp 158, Gln 155, Tyr 153, Tyr 229, Cys 228, His 227, Glu 226, Phe 203, Ser 202, His 200, Gln 179, His 174, Lys 167, Thr 166, Ser 165, Ser 164, Glu 163, and Phe 161 (Fig. 5D1–B).

### Physicochemical properties

The obtained results indicated that curcumin has a molecular weight (MW) of 368.38 g/mol, hydrogen bond doner (HBD) of 2, hydrogen bond acceptor (HBA)of 6, an oral bioavailability of 0.55, and Topological polar surface area (TPSA) value less than 2, as well as a high absorbance percentage of 93.06%, indicating that it has exceptional oral bioavailability (Table 4).

**Table 4:** Lipinski’s rule and in silico toxicity prediction of curcumin*.*

Ligand	AMES test	Carcinogenicity		hERG inhibition	Mw (g/mol)	Log p	HBA	HBD	TPSA (Å^2^)
		Mouse	Rat						
Curcumin	Non-mutagen	Negative	Positive	Medium risk	368.38	3.27	6	2	93.06

### Toxicity prediction

**Figure 6:**
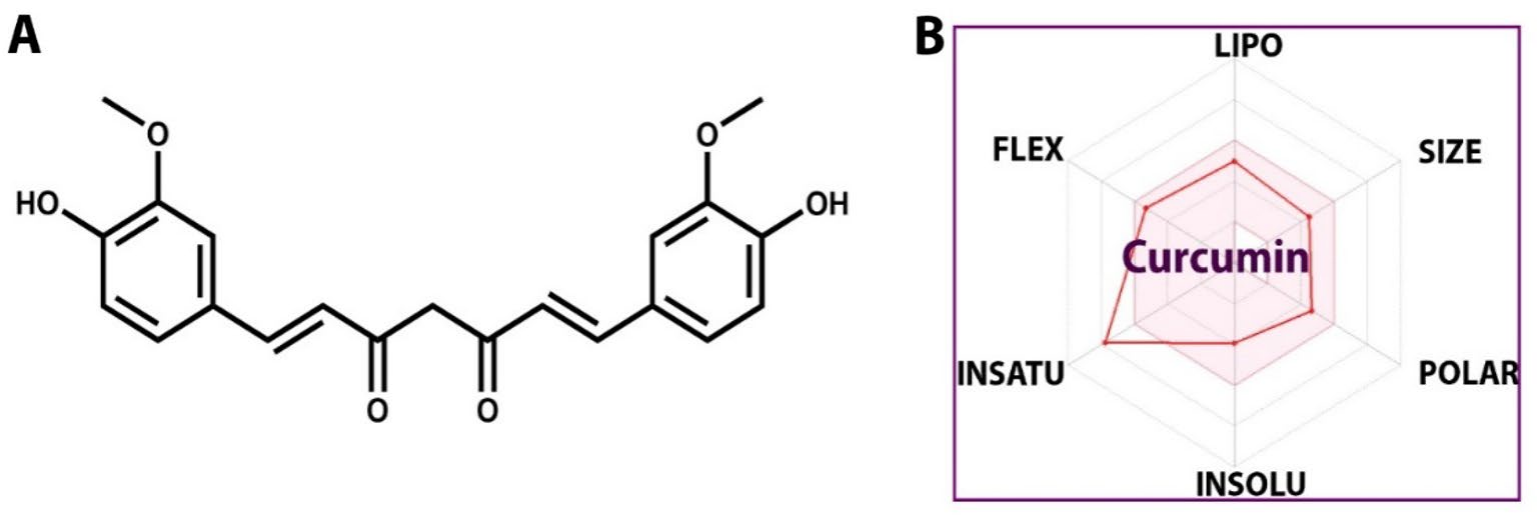
Structure of curcumin tested compound (**A**) and the ADME properties of the structure (**B**).

PreADMET was used to estimate the in-silico toxicity prediction of the ligand, and the results were displayed in Table 4. The curcumin molecule from drug bank structure (Fig. 6) was anticipated to be non-mutagenic according to AMES test. Furthermore, the compound's carcinogenicity in mice was expected to be negative. While the result was positive in case of rats, curcumin poses a modest risk according to the hERG test.

### Molecular dynamic (MD) simulation study of the proteins complexes with curcumin ligand

The results of the foregoing docking data prompted us to investigate the dynamic behaviour of SecA-SecY, LsrR, PqsR (MvfR) and AgrA bacterial quorum sensing pathway protiens in complex with curcumin, the root mean square deviation (RMSD), radius of gyration (Rg), polar surface area (PSA) and solvent accessible surface area (SASA) were all investigated and represented in Table 5. MD simulations of curcumin with four proteins from the four bacterial isolates (SecA-SecY from *B. subtilis*, LsrR from *E. coli*, PqsR (MvfR) from *P. aeruginosa*, AgrA from *S. aureus*) showed high binding affinity in every separated protein in relation to the binding sites of proteins RDB, the ligands were subjected to a 100 ns MD simulation to better understand the stability of the protein–ligand complexes.

**Table 5:** The Molecular dynamic simulation behavior of SecA-SecY, LsrR, PqsR (MvfR), and AgrA bacterial proteins in complex with curcumin ligand*.*

Bacterial protein	PDB ID	Ligand RMSD	MolSA	Rg	PSA	SASA
SecA-SecY	6itC	3	400	6.0	180	150
LsrR	4l5J	60	450	6.4	210	800
PqsR (MvfR)	6yiZ	3	375	5.2	180	100
AgrA	4g4K	3	395	6.0	190	450

*RMSD* root mean square deviation, *MolSA* molecular surface area, *Rg* the radius of gyration, *PSA* polar surface area, *SASA* solvent accessible surface area.

### Ligand–protein interaction root mean square fluctuation (RMSF) by md simulation study

**Figure 7:**
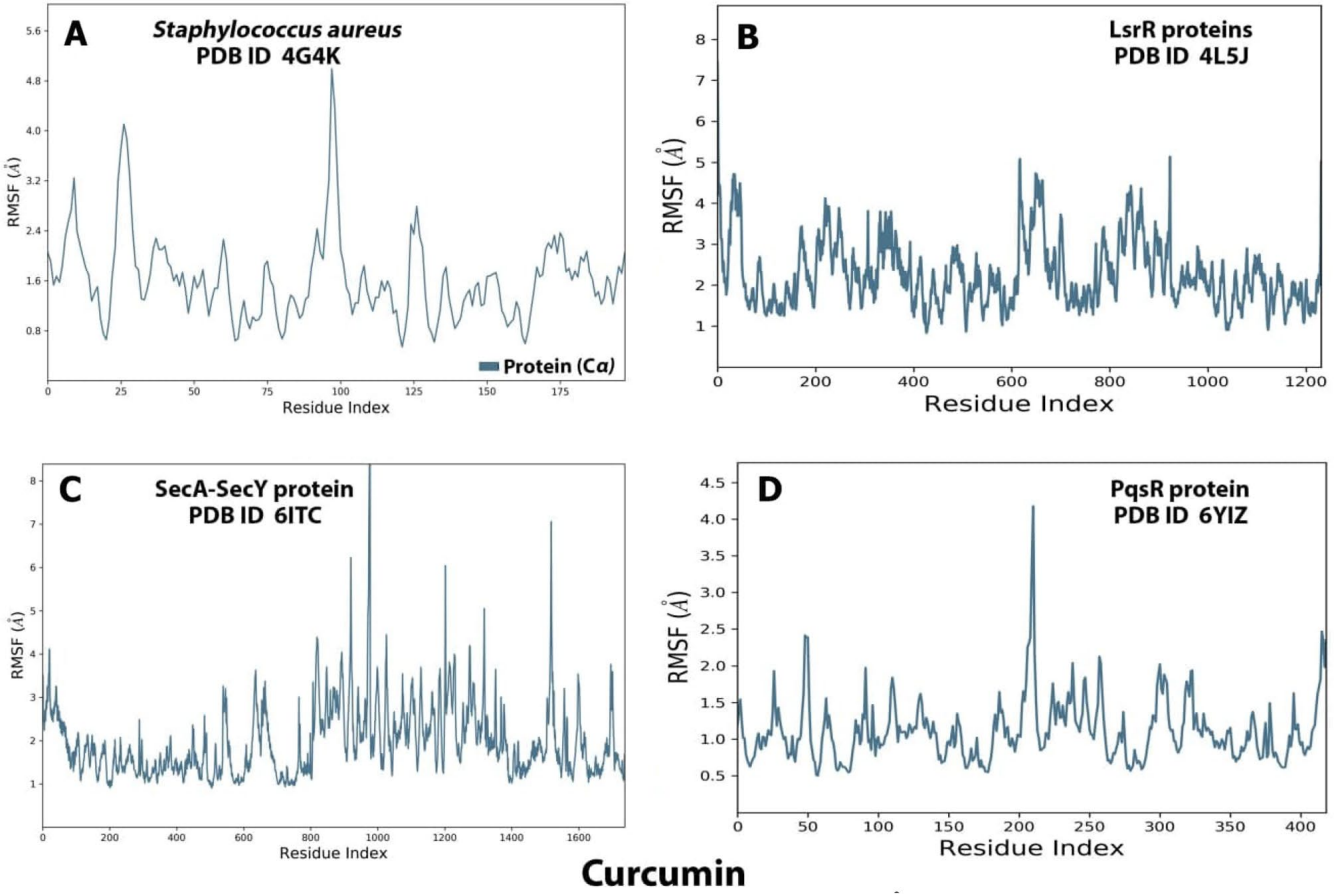
Root means square fluctuation (RMSF) (**A**) analysis of AgrA (**A**), LsrR (**B**), SecA-SecY (**C**), PqsR (MvfR) (**D**) proteins in association with curcumin complexes throughout 100 ns. SecA-SecY from *B. subtilis*, LsrR from *E. coli*, PqsR (MvfR) from *P. aeruginosa*, and AgrA from *S. aureus*.

MD simulation study demonstrated that AgrA (Fig. 7A) could effectively activate the biological pathway by modifying the conformation of the protein's C terminal and middle active site in the range of 0:50 to 75:125 respectively, on the other hand, the MD simulation of LsrR (Fig. 7B) revealed that it efficiently activates the biological pathway by changing the conformation in the C terminal and the middle of the protein between 0:200 residues in the C terminal and 600:900 residues in the middle of the protein, while the MD simulation of SecA-SecY (Fig. 7C) revealed that it efficiently activates the biological pathway with changes in conformation in all parts of the protein (C terminal, middle and N terminal in between 0:200 in C terminal, 800:1000 in the middle, and 1000:1600 in N terminal) residues, the MD simulation of PqsR (MvfR) (Fig. 7D) revealed that it effectively activates the biological pathway with changes in conformation in the middle and N terminal parts, with N terminal of the protein in between 400, and 175: 250 residues in the middle of the protein.

### Ligand–protein interaction root mean square deviation (RMSD) by MD simulation study

**Figure 8:**
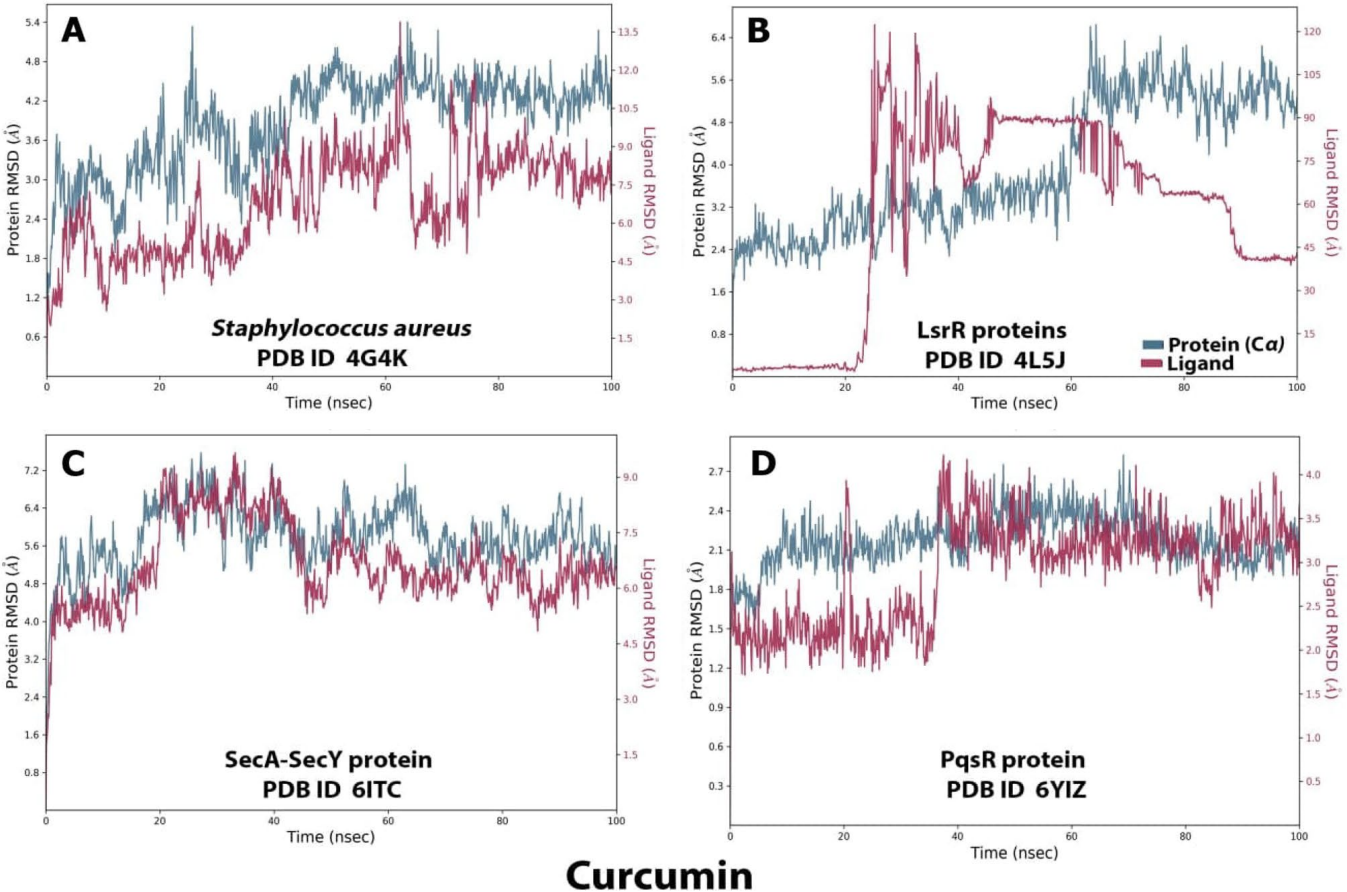
Root mean square deviation (RMSD) trajectories of AgrA (**A**), LsrR (**B**), SecA-SecY (**C**), and PqsR (MvfR) (**D**) protein complexes throughout 100 ns.[SecA-SecY from *B. subtilis*, LsrR from *E. coli*, PqsR (MvfR) from *P. aeruginosa*, and AgrA from *S. aureus*].

RMSD was used to evaluate the stabilities of protein (SecA-SecY, LsrR, PqsR (MvfR), AgrA) complexes with curcumin throughout 100 ns, it was used to measure the average change in displacement of a selection of atoms for a particular frame with respect to a reference frame. In this case, it was calculated for each frame of the trajectory. For AgrA the RMSD value for protein is 4.8 while 13.5 for ligand (Fig. 8A). For LsrR protein complex structure, the RMSD values were 6.4 and 170 for the protein and the ligand, respectively, which was not a good result (Fig. 8B), the SecA-SecY protein complex structure is depicted, the binding of the ligand and protein occur at 20 ns, the RMSD values were 7 and 9 for the protein and the ligand respectively (Fig. 8C). Furthermore, for the PqsR (MvfR) protein complex structure shown in Fig. 8D, the binding of the ligand and protein occurred during the first 100 ns, the protein's RMSD value was 2.7, while the ligand's RMSD value was 4. Also, the interaction of the ligand in the first 19 ns was less than 2.5, then increased to 3.5 at 20 ns, and then increased to around 4 in 100 ns.

### Protein–ligands contact plots and interactions

**Figure 9:**
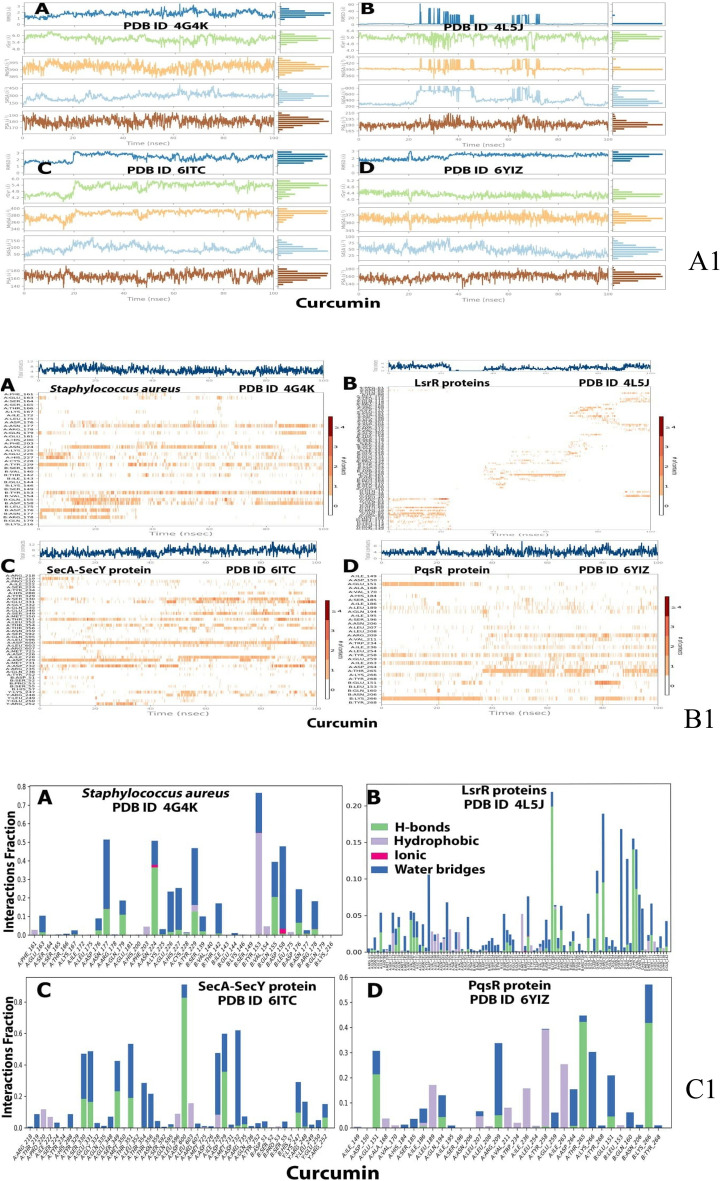
Ligand property trajectory (**A1**), protein–ligand plots (**B1**) and protein–ligand interaction residues (**C1**) for: AgrA protein (**A**), LsrR protein (**B**), SecA-SecY protein (**C**) and PqsR (MvfR) protein (**D**) during MD simulation at 100 ns.

The protein–ligand contact plots and interaction residues for AgrA were ASN 177, ASN 224, TYR 229, TYR 153, ASP158, and IEU 175, for LsrR there were no interacting residues, for SecA-SecY they were SER 330, GLU 331T It's LEU 189, LEU 189, GLN 194, TYP 258, ILE 263, THR 265, LYS 266 and LYS 266 , while for PqsR the interaction residues were Leu 189, Leu 189, Gln 194, Typ 258, Ile 263, Thr 265, Lys 266 and Lys 266. Hydrophobic, ionic, hydrogen bonds, and water bridges were the four types of P-L interactions identified (Table 6, Fig. 9).

**Table 6:** Binding energies (MMGBSA) of the complexes of matrix protein of RSV and the four selected bacterial proteins with curcumin.

Bacterial	Binding energy MMGBSA (kcal/mol)						
Proteins	dg bind	dg bind coulomb	dg bind covalent	dg bind H bond	dg bind lipo	dg bind solv gb	dg bind vdw
AgrA	− 41.1089	− 16.6193	2.415116	− 0.23531	− 13.2987	25.31413	− 38.6849
LsrR	− 26.84773396	− 12.8583	2.317894	− 1.25244	− 9.48339	23.67852013	− 29.2500197
SecA-SecY	− 59.26700736	− 28.6251	3.584246	− 3.60657	− 14.3359	31.431	− 47.7147
PqsR (MvfR)	− 54.8943	− 8.97489	3.327278	− 0.49142	− 19.8054	24.15725	− 53.1071

Dg; delta g. SecA-SecY from *B. subtilis*, LsrR from *E. coli*, PqsR (MvfR) from *P. aeruginosa*, and AgrA from *S. aureus.*

### In vivo evaluation of the infected wound healing model

**Figure 10:**
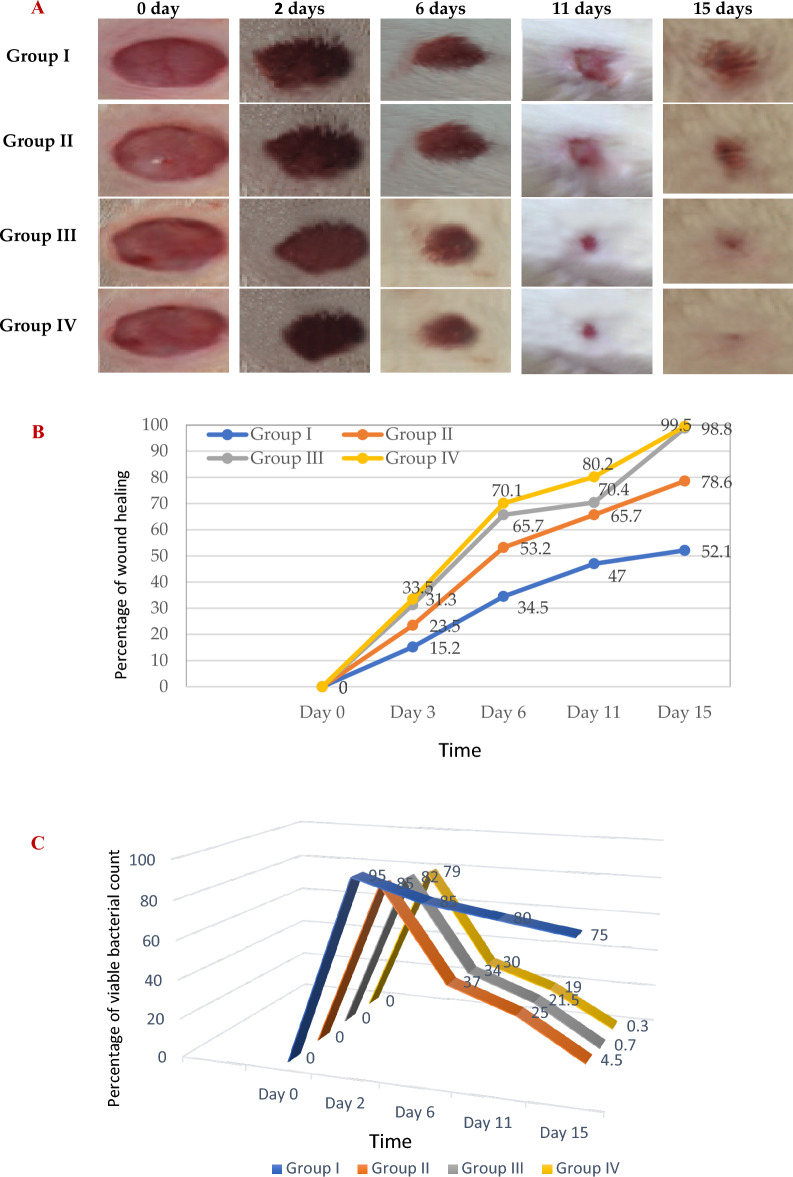
Wound healing over time (in days post-treatment) in vivo evaluation (**A**) photograph images, (**B**) the % of wound healing versus healing time, and (**C**) % bacterial viable count, results grouped in 20 s (0–20% blue, 20–40% orange, 40–60% gray, 60–80% yellow, and 80–100% green) in the studied animals, [Group I; infected wounded animals treated with blank lotion (negative control group), group II; infected wounded animals treated with micro-curcumin lotion, group III; infected wounded animals treated with nano-curcumin lotion, group IV; infected wounded animals treated with standard antibiotic Framycetin (Soframycin) ointment (positive control group).] [Animals per group = 6].

In this study 24 male albino rats were used, their dorsal thoracic central region was wounded, and infected by 10^6^ cfu *p. aeruginosa* to explore the antibacterial efficiency of topical treatment with MC and NC lotions in comparison to standard antibiotic Framycetin (Soframycin ointment), the last group left untreated (negative control group). Wound healing was evaluated after 3, 6, 11and 15 days, our findings presented in Fig. 10 showed induced time-dependent wound contraction and bacterial viability, along the treatment period (15-days). In group I (control) the healing percentage increased slowly to 52.1% on day 15 post-injury. Treating the infected wounded animal by the prepared MC lotion (group II) resulted in significant (*p* < 0.001) wound closure percentage compared to the control group I, reaching 78.6% at the 15th day post-wounding post-treatment. Remarkable improvement in wound closure was observed upon daily treatment of the third animal group (III) with NC lotion (*p* < 0.001), where the wound healing percentage was found to be 31.3% on the 2nd day post-injury and increased along the study entire duration, till reaching 98.8% wound closure on day 15-post wounding, post-treatment. Results shown were closely related to the positive control group treated with Soframycin (group IV). Reduction in the bacterial count was detected along treatment till it reached nearly 100% at the end of treatment period compared to the untreated group. The results obtained reflected the superiority of the daily usage of NC lotion as it had a better healing pattern and reduction in the time of wound contraction.

## Discussion

Curcumin is one of the traditional medicines which is widely used for biomedical applications^5^. To increase the productive use of curcumin, nanotechnology is considered a potential option, the intrinsic physicochemical characteristics of curcumin such as chemical instability, low bioavailability, and poor water limits pharmaceutical potential^10^, to overcome these drawbacks and improve the therapeutic use of curcumin nanotechnology is being considered a potential option. In this work Nanocurcumin particles were prepared via solvent-antisolvent precipitation which is quite attractive for its simplicity and affordability^40^, this method was carried out by dissolving MC into its solvent and then adding the antisolvent rapidly under constant stirring to reach super saturated condition, precipitation resulted in successful preparation of stable nanoparticles with irregular spherical shape and hydrodynamic diameter of 78.6 ± 8 nm, according to the national standard board guidance^41^, the size of the synthesized particles fall in the nano scale range compared with that of MC, curcumin nano formulation overcomes the challenge posed by its water insolubility as nano particles were found to be freely dispersed in water in the absence of any surfactants^42^, similar results were obtained by Dutta et al.^43^ who reported that curcumin nano formulation had improved its bioavailability and antimicrobial effects.

Numerous studies have reported the effectiveness of curcumin against a variety of microorganisms. This antimicrobial activity employs a multi-mechanistic strategy including membrane-nanoparticles interaction which causes local pores in the membrane and entry of nanoparticles that interact with the intra-cellular proteins, another possible mechanism is the binding of the nanoparticles to the bacterial membrane, and their gradual entry into the cytoplasm and disrupting the bacterial functions^16^, on the other hand the currently used antibiotics act by only one or few mechanisms, so they are subjected to microbial resistance^44^, In our study the in vitro antibacterial effect of both MC and NC were screened against standard Gram − ve and Gram + ve strains of the most prevalent wound infecting bacterial pathogens. Results obtained from both broth turbidity and TVC methods emphasized the previous findings which reported the antimicrobial potentiality of MC and NC preparations on a wide range of bacterial and fungal pathogens and the superiority of the nano particles preparation^45^. The susceptibility of the tested strains could be summarized in the order: *P. aeruginosa* > *B. subtilis* > *S. aureus* > *E. coli,* , these findings are in agreement with earlier reports of Gopal et al.^46^ and No et al.^47^ who declared that nanosized particles have better mobilization inside the cells better than their micro sized counterparts, the range of the minimum inhibitory concentration values on using NC suspension (15.65–31.25 µg/mL) was much lower than on using MC suspension (125–250 µg/mL) near results were obtained by Neto et al.^48^, Notably, there were significant differences in the MICs of curcumin against certain stains reported by different research groups . This may be due to the type of the solvent used by each research group^49^. Bacterial populations utilize a special chemical language in an autoinducer–receptor manner to regulate their virulence; this language is QS^50^. QS regulates biofilm formation, bacterial motility, and the production of virulent exocellular enzymes and pigments^51^, there are three main QS systems, (1) the acylhomoserine lactone (AHL) QS system in Gram-negative bacteria; (2) the autoinducing peptide (AIP) QS system in Gram-positive bacteria, and (3) the autoinducer-2 (AI-2) QS system, which is in both Gram-negative and Gram-positive bacteria^52^. The current in silico study gave us more detailed exploration about the inhibition activity of nanocurcumin indicated by the in vitro study, curcumin interaction with the selected four QS-encoding genes were evaluated, for *P. aeruginosa*, docking study of ligand–PqsR (MvfR) interaction was performed, PqsR (MvfR) is a critical transcriptional regulator with important roles in virulence, it can increase antibiotic efficacy and eventually prevent the AMR protein from forming^53^, for *B. subtilis* interaction was studied with SecA-SecY channel protein which permits a wide range of proteins to be transported across the eukaryotic endoplasmic reticulum membrane or across the prokaryotic plasma membrane, the SecA transports most secretory proteins post-translationally through the SecY channel in bacteria^18^. In *E. coli* ligand-LsrR interaction was performed, LsrR regulates hundreds of genes that participate in myriad biological processes, including mobility, biofilm formation, and antibiotic susceptibility and bacterial resistance to various compounds^20^, in case of *S. aureus* interaction was performed with AgrA transcription factor protein, which is involved in the regulation of the quorum-sensing response in the bacteria, as well as the generation of hemolysins and other virulence factors^51^. Docking scores indicated that the studied ligands were interacted with the protein's active site residues with energy binding affinity that varies from − 4.3 to − 7.8 kcal/mol, the values calculated for *P. aeruginosa* confirmed the high inhibitory effect of curcumin as it inhibited the active site of the PqsR protein with binding affinity of − 7.8 kcal/mol. The interaction results of SecA-SecY protein (*B. subtilis*), and AgrA protein (*S. aureus*) indicated moderate binding affinity − 6.6 and − 6.0 kcal/mol respectively, on the other hand the binding affinity of LsrR protein (5.5 kcal/mol) represent week interaction), the binding energy statistics were found to confirm the docking result. Curcumin has superior binding energies compared to other compounds^54^. RMSF was a good tool for assessing local alterations throughout the protein chain, while RMSD measures the average change in displacement of a particular frame relative to a reference frame for a sample of atoms, calculated for each frame along the path^28^. RMSD and RMSF studies were used to further examine the overall stability, where in case of PqsR protein the RMSD and RMSF values represent a stable interaction followed by Seca-Secy, and Agra proteins and finally the lowest values were for LsrR protein. In drug discovery and development, various tests were needed to determine whether the candidate drug is bioavailable and safe for the body or not. During drug-discovery pipeline and drug-development processes, various tests are utilized to determine whether the candidate/potential drug is bioavailable and safe for the body or not. Lipinski’s rule of five and toxicity tests are an example of criteria that need to be considered during this process. Nutraceuticals must meet four requirements (MW < 500, log P < 5, HBD ≤ 5, and HBA ≤ 10) to be classified as drug-like^55,56^. The resulting scores of curcumin predicted that it has exceptional oral bioavailability, also the physicochemical and structural characteristics of curcumin were inconsistent with most well-known drugs, and it didn’t meet all the toxicity tests. Wound bacterial infections are thought to play a serious role in healing delay by altering the host cell functions^57^, previous findings reported that the rate of infection is proportionally related to the number of inoculated bacterial colonies. Wound inoculation of 10^6^ cfu/mL resulted to 100% of the wounds without mortality, while increasing colony numbers to 10^10^ led to animals’ death, otherwise decreasing the number to 10^4^ approximately 50% of the wounds are showed no sign of infection^58^. Although concerns about using of antimicrobials on open wounds still exist because of their potential cytotoxicity that causes delay in the healing process^59^. In an application to evaluate the in vivo infected wound healing process, MC and NC lotions were prepared and applied topically on wounded rats once daily along 15 days from the first day of wounding, healing was observed by morphological examination and detected by calculating the healing percentage, it was found that treatment of the wounded animals by MC lotion significantly fastened the healing process compared to the control group, the healing percentage reached 78.6% on the fifteenth day of injury, similarly Dai et al.^60^ reported that curcumin facilitated complete wound reepithelization by reducing the epithelization period compared with the control group, also many studies have shown that topical application of curcumin to wound sites was more effective in the closure process^61^, this enhanced capacity of wound healing with the plant was based on its anti-inflammatory and antimicrobial effects that were well documented in the previous literature^62^, on the other hand, on treating the wound sites with the prepared NC lotion nearly complete wound closure was attained on the fifteenth day of treatment (98.8%). Earlier researchers declared that the in vivo wound treatment capability of curcumin nanoparticles was significantly higher than that of macro and micro curcumin particles, this must be attributed to the fact that its small size increases its interaction with the microbial and host cells^63^, also estimation of the total viable bacterial count indicated nearly 100% reduction at the end of the treatment interval. The obtained results revealed that the wounded animal group treated with NC lotion showed more well-formed granulation tissue and reepithelization earlier than other groups. Consequently, NC lotion could be an alternative strategy as a wound healing promotor.

## Conclusion

Abolishing bacterial growth requires developing new innovative approaches, obstructing the quorum-sensing bacterial pathway is one of them, according to the in vitro antibacterial assay nano-curcumin particles represent a significant advance for inhibiting both Gram-positive and Gram-negative bacteria. Moreover, the in-silico studies using four bacterial proteins, SecA-SecY, LsrR, PqsR (MvfR) and AgrA, affecting the quorum-sensing bacterial pathway revealed the efficacy of curcumin as a binding inhibitory ligand. Further studies based on molecular modeling showed that curcumin exhibit favorable docking scores making it in its nano form as the best candidate for the design of innovative formula. Superior results were noticed for *P. aeruginosa,* the most predominant nosocomial pathogen. In vivo antibacterial results pointed to the potential effectiveness of topical nano-curcumin lotion for decreasing the bacterial count percentage and enhancing wound healing activities.

### Study strength

Molecular modeling simulation proved the applicability of the concept nano-by-design “NbD” for curcumin topical formula, to serve as a topical therapy for wound infections and/or cutaneous injuries.

### Future prospects

Molecular mechanisms involved in wound environment hallmarks, are addressed both experimentally and computationally.

## Data Availability

The datasets used or analyzed during the current study are available from the corresponding author on reasonable request.
